# Cortex Moutan Induces Bladder Cancer Cell Death via Apoptosis and Retards Tumor Growth in Mouse Bladders

**DOI:** 10.1155/2013/207279

**Published:** 2013-10-24

**Authors:** Mei-Yi Lin, Ying-Ray Lee, Su-Yin Chiang, Yi-Zhen Li, Yueh-Sheng Chen, Cheng-Da Hsu, Yi-Wen Liu

**Affiliations:** ^1^Graduate Institute of Chinese Medicine, College of Chinese Medicine, China Medical University, Taichung 40402, Taiwan; ^2^Department of Chinese Medicine, Chiayi Christian Hospital, Chiayi 60002, Taiwan; ^3^Department of Medical Research, Chiayi Christian Hospital, Chiayi 60002, Taiwan; ^4^Department of Nursing, Min-Hwei College of Health Care Management, Tainan 73658, Taiwan; ^5^Department of Microbiology, Immunology and Biopharmaceuticals, College of Life Sciences, National Chiayi University, No. 300, Syuefu Road, Chiayi 60004, Taiwan; ^6^Department of Biomedical Imaging and Radiological Science, China Medical University, Taichung 40402, Taiwan

## Abstract

Cortex Moutan is the root bark of *Paeonia suffruticosa* Andr. It is the herbal medicine widely used in Traditional Chinese Medicine for the treatment of blood-heat and blood-stasis syndrome. Furthermore, it has been reported that Cortex Moutan has anticancer effect. In this study, the Cortex Moutan extract was evaluated in bladder cancer therapy in vitro and in vivo. Cortex Moutan extract reduces cell viability with IC_50_ between 1~2 mg/ml in bladder cancer cells, and it has lower cytotoxicity in normal urotheliums. It arrests cells in G1 and S phase and causes phosphatidylserine expression in the outside of cell membrane. It induces caspase-8 and caspase-3 activation and poly(ADP-ribose) polymerase degradation. The pan caspase inhibitor z-VAD-fmk reverses Cortex Moutan-induced cell death. Cortex Moutan also inhibits cell invasion activity in 5637 cells. In mouse orthotopic bladder cancer model, intravesical application of Cortex Moutan decreases the bladder tumor size without altering the blood biochemical parameters. In summary, these results demonstrate the antiproliferation and anti-invasion properties of Cortex Moutan in bladder cancer cells and its antibladder tumor effect in vivo. Cortex Moutan may provide an alternative therapeutic strategy for the intravesical therapy of superficial bladder cancer.

## 1. Introduction

Cortex Moutan (CM, root bark of *Paeonia suffruticosa* Andr.), named Mu Dan Pi in Chinese, is the herbal medicine widely used in Traditional Chinese Medicine (TCM). It tastes bitter and pungent. According to TCM theory, it belongs to the light cold-type medicine and has the function of heat-clearing, promoting blood circulation, removing blood stasis, cooling the blood, and reducing the deficient heat. In clinical regimen, CM is usually used to treat heat in the blood and blood stasis syndromes. It is used as an analgesic [1], antispasmodic, antiaggregatory [2, 3], and antioxidative agent [4]. In other reports, CM itself or its major component has been shown to treat various disorders like diabetes [5], Alzheimer's disease [6, 7], arthritis [8], inflammation [9, 10], sepsis [11], brain ischemia-reperfusion injury [12], HIV infection [13], and herpes simplex virus infection [14]. Recently, there are also some reports showing the anitumor activity of CM, including renal carcinoma [15], DLD-1 human colon cancer cells [16], and gastric cancer [17]. In addition, one component of CM, paeoniflorin, was reported to have antitumor effect through apoptosis in lung cancer cells [18].

Bladder cancer is a common malignancy worldwide [19]. A person with the history of urinary tract cancer in first-degree relatives and heavy cigarette smoking increases the risk of bladder cancer [20]. More than 90% of bladder cancers are transitional cell carcinoma (TCC) in the histology, and it is classified into two groups in the pathogenesis: nonmuscle invasive and muscle invasive [21]. There are two groups of intravesical therapeutic agents for bladder cancer therapy. Bacillus Calmette-Guérin (BCG) is the immunotherapeutic agent, and mitomycin C is one of the chemotherapeutic agents [21, 22]. Up to now, BCG provokes considerable and sometimes serious side effects, and the recurrence rate remains high in spite of intravesical chemotherapy. There is certainly place for improvement in bladder cancer therapy. Intravesical antitumor herbal medicine may provide an alternative pathway in the treatment of TCC.

The antitumor effect of CM extract on bladder cancer is still unknown. In this study, we investigated the anticancer effect of CM extract in two bladder cancer cell lines and the in vivo effect in a mouse orthotopic bladder tumor model.

## 2. Materials and Methods

### 2.1. Cell Culture and CM Extract Preparation

Human bladder papillary transitional cell carcinoma 5637 cells were obtained from the Bioresource Collection and Research Center (Hsinchu, Taiwan). Mouse bladder carcinoma MB49 cells were kindly provided by Dr. Timonthy L. Ratliff (Purdue Cancer Center, West Lafayette, IN). 5637 and MB49 cells were maintained in RPMI 1640 medium supplied with 10% fetal bovine serum (FBS), 1% penicillin, and 1% streptomycin. Cells were incubated in a CO_2_ incubator at 37°C, with 5% CO_2_ and 95% filtered air. CM powder was purchased from Sun Ten Pharmaceutical Co., Ltd. (Taipei, Taiwan). CM extract was prepared by mixing CM powder with RPMI 1640 medium (50 mg/mL), sonicated on ice for 60 min, filtered by the qualitative filter paper, 0.45 *μ*m filter and 0.2 *μ*m filter, then stocked at −80°C before use. The stock concentration is 50 mg/mL, and RPMI 1640 medium is used for dilution.

### 2.2. Reagents and Antibodies

Propidium iodide (PI), ribonuclease A (RNase A), z-VAD-fmk, and formaldehyde were purchased from Sigma (St. Louis, MO, USA). Annexin V-FITC apoptosis detection kit was purchased from Strong Biotech Corporation (Taipei, Taiwan). Antibodies against caspase-8 (D35G2), cleaved caspase-3 (Asp175), and poly(ADP-ribose) polymerase (PARP) were purchased from Cell Signaling (Danvers, MA, USA). Anti-glyceraldehyde-3-phosphate dehydrogenase (GAPDH) antibody was purchased from GeneTex (Taichung, Taiwan). The Millicell Hanging Cell Culture Inserts of Transwell system was purchased from Millipore (Billerica, MA, USA).

### 2.3. Cell Viability Assay

Cell number was determined by direct cell counting. MB49 (4 × 10^4^ cells/well) or 5637 (5 × 10^4^ cells/well) cells were cultured in 24-well plates; SV-HUC1 cells (2 × 10^4^ cells/well) were cultured in 96-well plate. After 24 h, cells were incubated with various concentrations of CM extract for another 24 and 48 h. The cells were detached by trypsin treatment, trypan blue staining, and counted by a haemocytometer. The result was expressed as a percentage, relative to control group.

### 2.4. Cell Cycle Analysis

MB49 (1 × 10^6^ cells/dish) or 5637 (2 × 10^6^ cells/dish) cells were seeded in 100 mm dishes. After 24 h incubation for attachment, CM extract was added. After treatment for 24 h and 48 h, cells were trypsinized, centrifuged, and fixed with ice-cold 75% ethanol overnight at 4°C. After removing the ethanol, cells were stained with a DNA staining solution (containing 1 mg/mL PI and 10 mg/mL RNase A dissolved in PBS) for 30 min at room temperature. The DNA content of the stained cells was measured using a FACScan flow cytometer. The cell doublets were removed by gating the left area of FL2-W/FL2-A plot for analysis. Cell cycle data from flow cytometry was analyzed using ModFit LT software which distinguished cell cycle to G1 (2n), S (between 2n to 4n), G2/M (4n), and sub G1 (debris) according to DNA content in a single cell.

### 2.5. Apoptotic Cell Death Analysis

MB49 (1 × 10^6^ cells/dish) or 5637 (2 × 10^6^ cells/dish) cells were seeded in 100 mm dishes. After 24 h, CM extract was added. After treatment for 24 h and 48 h, cells were collected after washing, trypsinization, and centrifugation. The cell pellets were resuspended in 100 *μ*L of staining solution (2 *μ*L Annexin-V-FITC and 2 *μ*L PI in 100 *μ*L binding buffer) and incubated for 15 min at room temperature. Annexin-V or PI fluorescent intensities were analyzed by a FACScan flow cytometer.

### 2.6. Cell Invasion Assay

The invasion assay was analyzed using a Matrigel- (BD Biosciences) coated Transwell system (Millipore). The upper chamber of the transwell was coated with 25 *μ*g Matrigel. 5637 cells (1 × 10^5^) in serum-free RPMI-1640 media were seeded onto Matrigel-coated Transwell. CM extract was added into upper and lower chamber media. In the lower chambers, 10% FBS was added as a chemoattractant. After a 24 h incubation time, the cells that remained on the upper surface of the filter membrane were removed, and the cells on the opposite surface of the filter membrane were fixed with 4% formaldehyde for 30 s, stained with crystal violet and photographed under microscopy at 200x magnification. The number of migrated cells was counted in five randomly chosen microscope fields.

### 2.7. Mouse Orthotopic Bladder Tumor Model

The female C57BL/6 mice aged six weeks were provided by the National Laboratory Animal Center (Taipei, Taiwan) and maintained at our animal care facility for one week prior to use. The implantation of mouse bladder cancer cells MB49 into C57BL/6 mice was carried out similarly as previous reports [23–25]. After MB49 inoculation (Day 1), mice were randomly assigned to two groups (8 mice/group). One group was intravesically treated with RPMI 1640 medium, and the other group received 2.5 mg/mouse CM extract intravesically every other day from Day 16 to 24. At the 26th day, the mice were sacrificed, and the bladder volumes were measured before formalin fixation. After cutting the paraffin-embedded bladder tissues into 4 *μ*m sections, the slides of each mouse bladder were confirmed under a microscope in histology by hematoxylin and eosin staining. The experiment was approved by the Institutional Animal Care and Use Committee of National Chiayi University.

### 2.8. Statistical Analysis

The values shown are mean ± SEM. Data are statistically evaluated by one way ANOVA and shown significantly different in *P < 0.05, **P < 0.01, and ***P < 0.001.

## 3. Results

### 3.1. Cytotoxicity of CM Extract in MB49, 5637, and SV-HUC1 Cells

**Figure 1:**
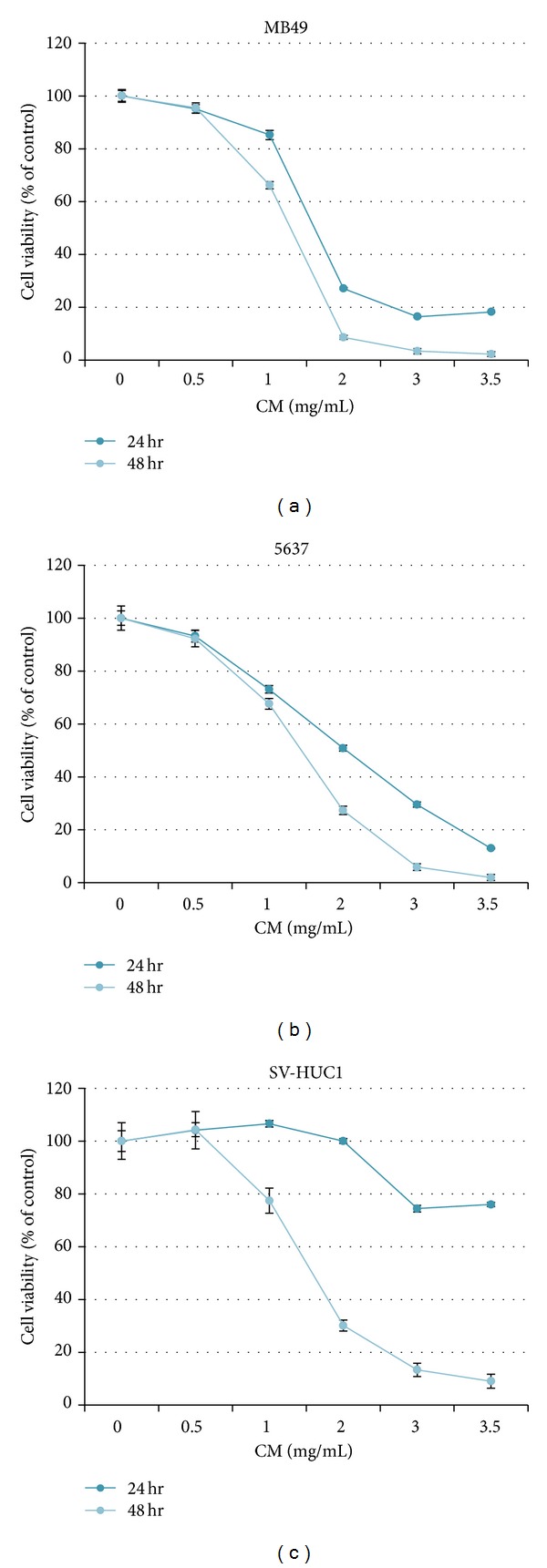
The cytotoxicity of CM extract in bladder cancer cells and normal urotheliums. The cytotoxicity was analyzed after CM treatment for 24 h and 48 h. The cell number of control was regarded as 100%. Data represent the mean ± SEM of quadruplicate. The experiment was repeated for three times.

In mouse bladder cancer cells MB49, the IC_50_ of CM extract is 1.3 mg/mL at 48 h treatment and 1.6 mg/mL at 24 h (Figure 1(a)). In human bladder cancer cell 5637, the IC_50_ of CM extract is 1.4 mg/mL and 2.0 mg/mL at 48 h and 24 h treatment, respectively (Figure 1(b)). In human normal urothelium SV-HUC1 cells, the IC_50_ is 1.6 mg/mL at 48 h, but it is higher than 3.5 mg/mL at 24 h (Figure 1(c)). According to these data, CM extract shows a good anticancer effect and a minor damage to normal cells at 24 h treatment.

### 3.2. CM Extract Induces Apoptotic Cell Death

**Figure 2:**
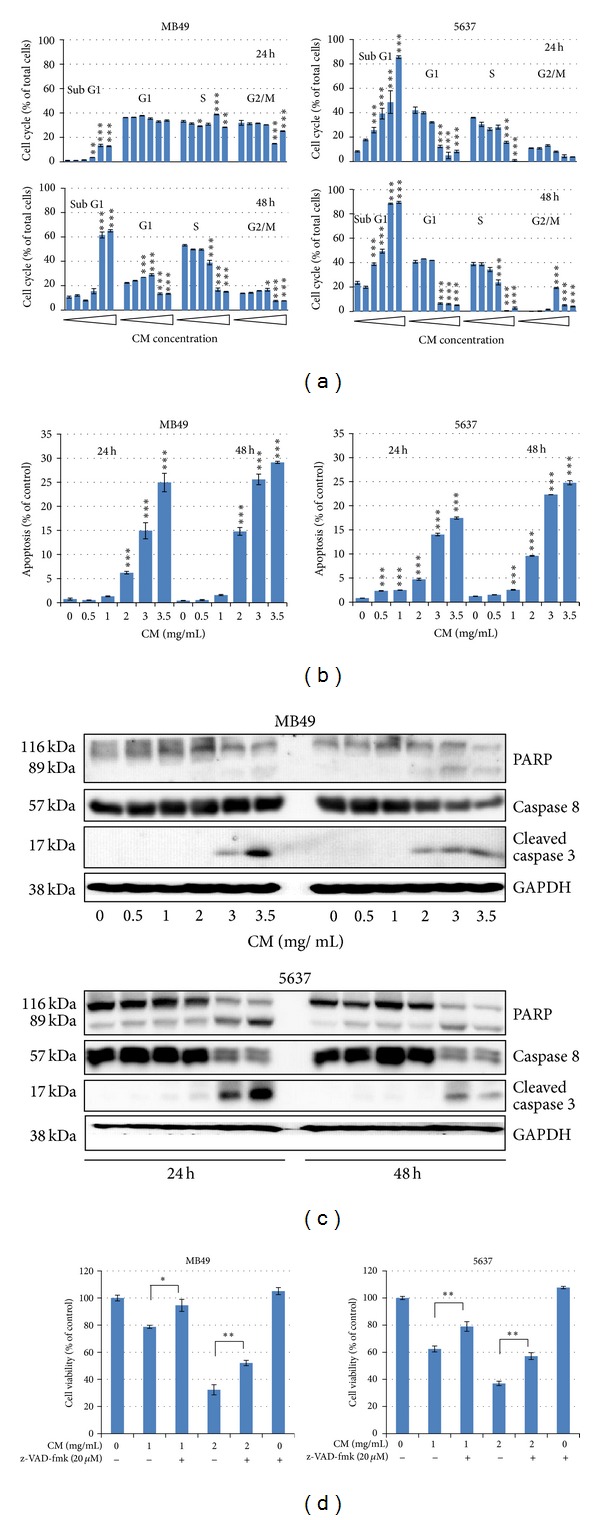
CM extract induces cell apoptosis. (a) Cell cycle distribution after CM treatment. Cells were treated with medium or CM extract (0, 0.5, 1, 2, 3, 3.5 mg/mL) for 24 h and 48 h then collected for cell cycle analysis. Data represent the mean ± SEM of triplicate. *P < 0.05, **P < 0.01, and ***P < 0.001 compared with control. (b) Apoptosis analysis by Annexin-V-PI staining assay. The apoptosis percentage implied the Annexin-V-positive and PI-negative staining cells. Data represent the mean ± SEM of triplicate. ***P < 0.001 compared with control. (c) Western blot analysis of apoptotic proteins including PARP (original 116 kDa and degraded 89 kDa forms), original caspase-8, activated caspcas-3, and GAPDH (internal control). (d) z-VAD-fmk reverses CM-induced cell death. z-VAD-fmk was added in medium 1 h before CM treatment, and cell number was counted after CM treatment for 24 h. Data represent the mean ± SEM of quadruplicate. *P < 0.05, and **P < 0.01 compared with each other. All the experiments were repeated for three times.

Cell cycle analysis was performed in MB49 and 5637 cells after CM extract treatment. CM dose dependently increased sub-G1 population while decreasing G1 and S population in MB49 and 5637 cells (Figure 2(a)). CM also increased exposed phosphatidylserine by Annex V-FITC staining assay in MB49 and 5637 cells (Figure 2(b)). The CM-induced apoptosis was also confirmed by Western Blot analysis. It shows that CM dose dependently induces the activation of caspase-8 and caspase-3, and degrades PARP in MB49 and 5637 cells (Figure 2(c)). When the pan-caspase inhibitor z-VAD-fmk was added, it partially reversed CM-induced cell death (Figure 2(d)). These results suggest that CM induces cell death at least via extrinsic apoptosis pathway, which was concurrent with the activation of caspase-8 and caspase-3.

### 3.3. CM Extract Inhibits Cell Invasion in 5637 Cells

**Figure 3:**
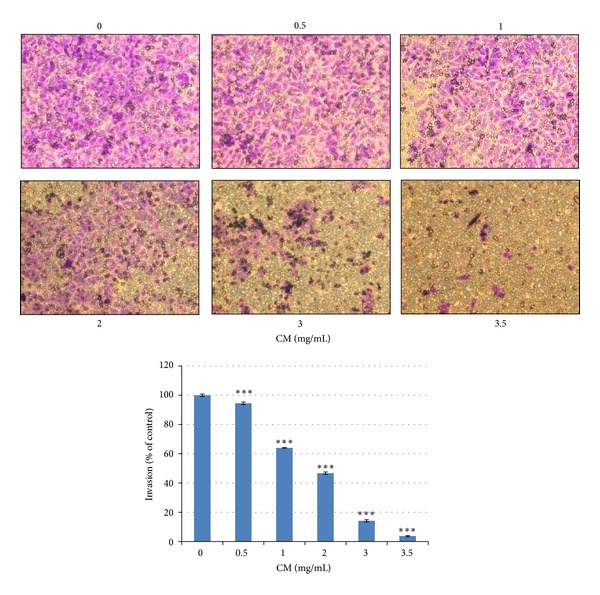
CM extract inhibits cell invasion. After medium or CM treatment for 24 h, migrated 5637 cells were counted. The cell number of control was regarded as 100%. Data represent the mean ± SEM of triplicate. ***P < 0.001 compared with control. The experiment was repeated for three times.

The cell invasion assay was analyzed by Matrigel-coated transwells. The invasive activity of 5637 cells is stronger than that of MB49 cells, so the assay is explored in 5637 cells only. The data indicates that CM extract dose dependently inhibits cell invasion, and the inhibition percentage is higher than that of cell growth at the same dose (Figure 3). It suggests that CM extract, besides cytotoxicity, also have anti-invasive activity.

### 3.4. CM Extract Inhibits Tumor Growth in a Mouse Orthotopic Bladder Tumor Model

**Figure 4:**
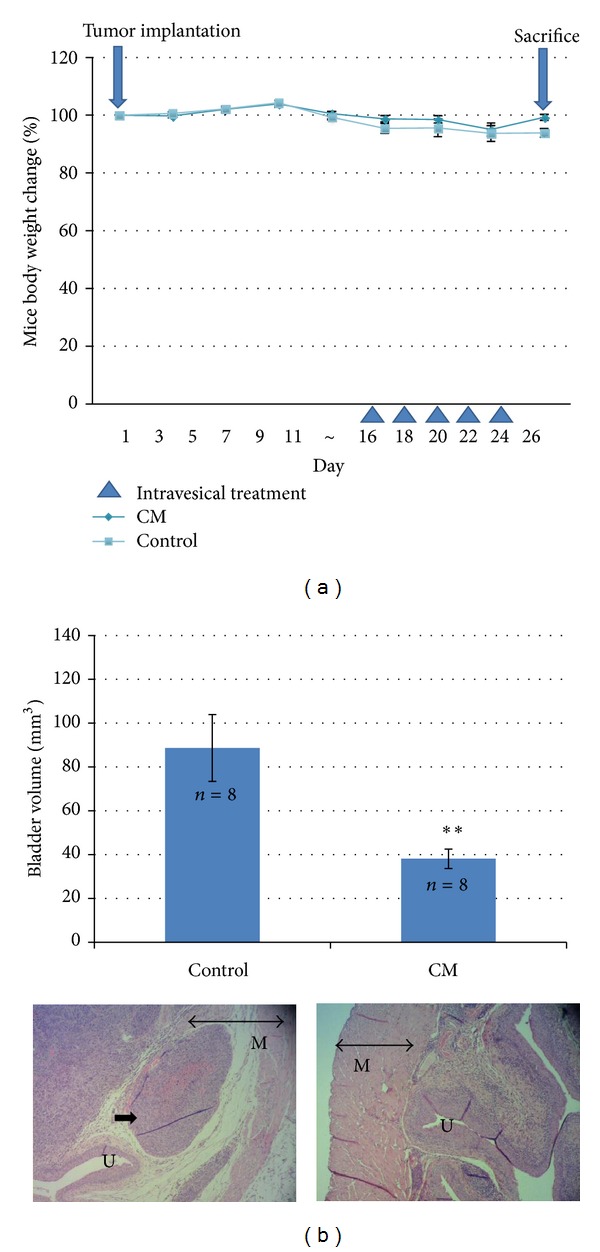
The antitumor effect of CM extract in a mouse orthotopic bladder tumor model. (a) Mouse body weight and drug schedule. MB49 cells were implanted at Day 1. Mouse body weight was recorded weekly or every operation. (b) Intravesical application of CM extract retards mouse bladder tumor growth and muscle invasion. After tumor implantation, CM extract was applied intravesically for 5 times. After sacrifice, each bladder volume (mm^3^) was calculated by (length × wide^2^)/2. Data represent the mean ± SEM. **P < 0.01 compared with control. In the HE stain slide photos, M: muscle, U: urothelium, double-head arrow: muscle layer, and single arrow in control: the tumor invading into muscle layer.

After tumor implantation at Day 1, CM extract intravesical application was developed from Day 16 to Day 24 once two days. The body weights did not significantly change between control and CM-treated mice (Figure 4(a)). The bladder size was calculated after sacrifice at Day 26. Intravesical CM treatment significantly decreased bladder volume and retarded tumor invading into muscle layer (Figure 4(b)). In the blood biochemistry data, it shows no significant difference between 2 groups (Table 1). It suggests that intravesical CM treatment decreases bladder tumor size without adverse event in liver and kidney.

## 4. Discussion

This study provides the first evidence that CM extract has antibladder cancer activity via extrinsic apoptosis pathway (Figure 2) and reduces mouse bladder tumor size using intravesical therapy (Figure 4). In addition to the therapeutic effect, CM also shows a good distinctive effect between normal bladder and cancer cells (Figure 1). After mouse intravesical therapy for 5 times, there is no harm by physiological observation and no damage to liver and kidney by blood biochemical parameter analysis (Table 1). This data suggest that CM extract might provide an alternative therapeutic agent for bladder cancer.

The aqueous extract of *Paeonia suffruticosa* has been reported to inhibit renal cancer cell invasion and metastasis via VEGFR-3 suppression [15]. In this study, we also prove that CM extract has anti-invasion effect in 5637 cells (Figure 3). Some bioactive anticancer compounds have been identified from *Paeonia suffruticosa*. For example, paeonol has anticancer activity in esophageal cancer cells [26] and liver cancer cells [27]; paeoniflorin inhibits lung cancer cells growth via apoptotic cell death [18]. In addition to these two compounds, there are 50 compounds found in 50% (v/v) methanol extract of *Paeonia suffruticosa* including 17 monoterpenes, 14 galloyl glucose, 10 acetophenones, 5 phenolic acids, 3 flavonoids, and 1 triterpene [28]. The active compound(s) for antibladder cancer activity need be investigated in the future. In this study, CM extract induces extrinsic apoptosis to cause caspase-8 activation and cell death (Figure 2(c)). This phenomenon is also reported by other reports. The ethanol extract of *Paeonia suffruticosa* [17] and paeoniflorin [18] induce Fas/Fas ligand-mediated extrinsic apoptotic cell death.

Mouse orthotopic bladder tumor is a good model to mimic human bladder tumor and could be applied intravesically, which is the most widely used therapy for superficial bladder tumor [29]. In our previous studies, intravesical application of nature compound combretastatin A-4 also provides a good therapeutic effect but with some adverse events in blood parameters [23]; intraperitoneal baicalein shows a little effect in reducing bladder tumor size [25]. CM extract is the mixture of some bioactive compounds, but it shows a benefit in bladder cancer intravesical therapy. In conclusion, the present data elucidate the antibladder cancer effect of CM extract in vitro and in vivo and suggest that CM extract may provide an alternative therapeutic strategy for the intravesical treatment of superficial bladder cancer.

## Figures and Tables

**Table 1:** The blood biochemical parameters after CM extract intravesical application. After sacrifice, the blood was collected for preparing serum which was provided for biochemical parameter analysis.

Biochemical parameters	Control	CM
BUN (mg/dL)	25.50 ± 1.22	24.35 ± 1.02
Creatine (mg/dL)	0.56 ± 0.03	0.57 ± 0.02
SGOT (U/L)	192.93 ± 51.97	217.90 ± 28.91
SGPT (U/L)	28.0 ± 2.75	28.87 ± 2.20

Data represent the mean ± SEM. There are no significant differences between control and CM-treated groups.

## Abbreviations

BCG: Bacillus Calmette-Guérin
CM: Cortex Moutan
FBS: Fetal bovine serum
GAPDH: Glyceraldehyde-3-phosphate dehydrogenase
PARP: Poly(ADP-ribose) polymerase
TCC: Transitional cell carcinoma
TCM: Traditional Chinese Medicine
VEGFR: Vascular endothelial growth factor receptor.
